# Sexueller Missbrauch im Verantwortungsbereich der Evangelischen Kirche: Einige Ergebnisse aus einem Teilprojekt der ForuM-Studie

**DOI:** 10.1007/s00115-024-01670-0

**Published:** 2024-04-29

**Authors:** Harald Dreßing, Dieter Dölling, Elke Voss, Leonie Scharmann, Andreas Hoell

**Affiliations:** 1 Klinik für Psychiatrie und Psychotherapie, Zentralinstitut für Seelische Gesundheit, Medizinische Fakultät Mannheim, Universität Heidelberg, J 5, 68159 Mannheim, Deutschland; 2 Institut für Kriminologie, Universität Heidelberg, Heidelberg, Deutschland

## Hintergrund

Nachdem die Evangelische Kirche in Deutschland (EKD) ein Forschungsvorhaben zur „Aufarbeitung von sexualisierter Gewalt und anderen Missbrauchsformen in der Evangelischen Kirche und Diakonie in Deutschland“ öffentlich ausgeschrieben hatte, kam es zu einem kompetitiven Bewerbungsverfahren, an dem sich mehrere Forschungskonsortien beteiligten. Es bekam ein Konsortium den Zuschlag, an dem WissenschaftlerInnen unterschiedlicher Fachrichtungen der Hochschule Hannover, der Forschungsstelle für Zeitgeschichte Hamburg, der Bergischen Universität Wuppertal, der Fachhochschule Potsdam, des Instituts für Praxisforschung und Projektberatung München, des UKE Hamburg, des Zentralinstituts für Seelische Gesundheit Mannheim und des Kriminologischen Instituts der Universität Heidelberg beteiligt waren. Die Studie umfasste ein Metaprojekt sowie fünf Teilprojekte mit unterschiedlichen methodischen Zugängen. Im Oktober 2020 erfolgte die Zuwendungsvereinbarung zwischen der EKD und den Teilprojekten, die wissenschaftliche Arbeit startete mit Beginn 2021.

Teilprojekt E, das von den Autoren dieses Artikels verantwortlich geleitet wurde, befasste sich mit der Ermittlung von Kennzahlen zur Häufigkeit des sexuellen Missbrauchs im Bereich der evangelischen Kirche sowie mit dem Umgang der Institution mit Missbrauchsvorwürfen. Über die Daten dieses Teilprojekts sowie die Schwierigkeiten in der Zusammenarbeit der Wissenschaftler mit den Landeskirchen bei der Durchführung dieses Teilprojekts wurde in den Medien ausführlich berichtet. Aus diesem Grund werden im Folgenden einige zentrale Ergebnisse des Teilprojekts E dargestellt.

## Methodik

Im Teilprojekt E waren – vergleichbar dem erfolgreich praktizierten Vorgehen bei der MHG-Studie, in der der sexuelle Missbrauch im Verantwortungsbereich der katholischen Kirche untersucht worden war [2] – zwei Teilschritte geplant. In Teilschritt 1 wurde ein umfangreicher Fragebogen an alle 20 Gliedkirchen der EKD versandt, mit dem Informationen u. a. zu folgenden Themenkomplexen ermittelt wurden: Regelungen und Praktiken im Umgang mit Hinweisen auf sexualisierte Gewalt, Archivierungsregeln und -praxis von Verdachtsfällen, Kennzahlen zu bereits bekannten Fällen der Landeskirchen und Diakonischen Werke, Umsetzung von Präventionsmaßnahmen, Schulungen und Fortbildungen zum Thema.

In Teilschritt 2 sollten zunächst die bereits bekannten Fälle systematisch in einem Erfassungsbogen detailliert analysiert werden in Hinblick auf Merkmale von Beschuldigten, Betroffenen sowie der Tatkomplexe und der Tatfolgen. Daran anschließend war mit der EKD eine Personalaktenanalyse von Beschäftigten der Landeskirchen vereinbart, um Hinweise für bisher nicht bekannte Fälle zu ermitteln.

## Ergebnisse

Neben der Darstellung einiger inhaltlicher Ergebnisse ist es notwendig, kurz auf den Ablauf der Studie und die Probleme in der Zusammenarbeit mit den Landeskirchen einzugehen, da sich hieraus auch qualitative Hinweise auf den Stellenwert der Thematik in der EKD ergeben. Im ersten Teilschritt war die Zuarbeit der Landeskirchen schleppend, und es wurden teilweise qualitativ ungenügende Informationen übermittelt. Das führte zu einer massiven zeitlichen Verzögerung von über einem Jahr, was eine Änderung im vorgesehenen Forschungsdesign notwendig machte. Die ursprünglich vorgesehene umfangreiche Personalaktenanalyse war aus zeitlichen Gründen nicht mehr möglich, sodass man sich mit der EKD im Verlauf der Studie auf eine Durchsicht der Disziplinarakten von Pfarrpersonen einigte, um ein vollständiges Scheitern des Projekts zu verhindern. Von der kommissarischen Ratsvorsitzenden der EKD wurde das Verhalten der Landeskirchen bei der öffentlichen Vorstellung der Ergebnisse der Studie auf einer Pressekonferenz nicht als „ein bewusstes Nicht-Wollen, sondern ein an vielen Stellen unglückliches Nicht-Können“ bezeichnet, den Landeskirchen sei der Umfang der zu leistenden Zuarbeit erst im Verlauf der Studie deutlich geworden [5]. Einige Landeskirchen stellten im Nachhinein sogar in Abrede, dass eine Personalaktenanalyse mit den Wissenschaftlern vereinbart gewesen sei, was die EKD veranlasste, klarzustellen, dass eine solche Personalaktenanalyse vertraglich durchaus vereinbart worden war [4].

Obwohl die Beschränkung auf die Analyse der Disziplinarakten eine erhebliche Selektion der Datenbasis darstellt, konnte doch eine nicht unerhebliche Zahl von sexualisierten Gewalthandlungen zum Nachteil von Kindern und Jugendlichen im Verantwortungsbereich der EKD ermittelt werden. Es konnten anonymisierte Daten zu 1259 Beschuldigten, davon 511 Pfarrpersonen, und 2225 Betroffenen erhoben werden. Bis auf eine beschuldigte Person waren die beschuldigten Pfarrpersonen alle männlichen Geschlechts. Abhängig von der jeweiligen Quelle betrug das durchschnittliche Alter der Betroffenen bei den bekannten Fällen der Landeskirchen 11,7 Jahre, wobei 52,5 % der Betroffenen männlich waren, bei den Fällen der Diakonischen Werke 11,1 Jahre, davon 81,6 % männlich, und bei den aus den Disziplinarakten ermittelten Fällen 13,4 Jahre, davon 44 % männlich. Die Taten waren überwiegend geplant. Die Tatkontexte waren sehr unterschiedlich und bestanden z. B. in der Gemeindearbeit, dem evangelischen Pfarrhaus, Jugendfreizeiten, Heimen und Bildungseinrichtungen. In den meisten Fällen kam es zu mindestens einer Hands-on-Handlung, wobei die Tathandlungen hinsichtlich der Schwere eine große Spannweite aufwiesen und von verbalen Ansprachen mit sexuellem Inhalt bis zur analen und genitalen Penetration sowie oralem Verkehr reichten. Eine detaillierte Darstellung aller Ergebnisse findet sich im umfangreichen Abschlussbericht [1].

In einer kleineren Landeskirche war es noch möglich, eine vollständige Durchsicht aller Personalakten von Pfarrpersonen durchzuführen. Dabei zeigte sich, dass eine systematische Durchsicht der Personalakten einen erheblichen zusätzlichen Erkenntnisgewinn erbringen kann. In dieser Landeskirche wären nämlich bei einer ausschließlichen Disziplinaraktenanalyse 57,1 % der Beschuldigten und 73,8 % der Betroffenen nicht erfasst worden. Eine systematische Durchsicht aller Personalakten in den anderen Landeskirchen ist vor diesem Hintergrund nicht nur ein Forschungsdesiderat, sondern eine unabdingbare Voraussetzung, um eine transparente Aufarbeitung des Missbrauchsgeschehens in der EKD zu beginnen.

## Diskussion

Die Ergebnisse zeigen, dass sexualisierte Gewalt zum Nachteil von Kindern und Jugendlichen auch im Verantwortungsbereich der EKD ein erhebliches Problem ist.

Dabei haben sich die föderale Struktur der EKD und die komplexe Verwaltungsorganisation, die mit der Gefahr einer Verantwortungsdiffusion und Verantwortungsdelegation verbunden sein können, als spezifisch evangelische Risikofaktoren bei dieser Thematik erwiesen. Dies hat sich nicht nur beim oft wenig sensiblen Umgang der EKD mit den Betroffenen gezeigt – bei dem der Schutz der Institution oft vor die Bedürfnisse der Betroffenen gestellt wurde –, sondern auch in der Zusammenarbeit der Landeskirchen mit den Forschenden bei dieser Studie. Als ein weiterer spezifischer Risikofaktor kann das evangelische Pfarrhaus als ein Tatort benannt werden, weil sich hier in besonderem Maß die Gefahr einer Vermischung von Privatem und Dienstlichem ergeben kann, was eine besondere Ermöglichungsstruktur von Missbrauchshandlungen darstellt.

Das Selbstbild der EKD als die „bessere Kirche“, verbunden mit dem Narrativ, dass sexualisierte Gewalt vornehmlich ein Problem der katholischen Kirche sei, bedarf im Licht dieser Studienergebnisse jedenfalls einer kritischen Analyse.

Bei der Interpretation der Befunde ist zu berücksichtigen, dass in Deutschland insbesondere die beiden großen christlichen Kirchen umfangreiche Studien zu sexualisierter Gewalt in Institutionen in Auftrag gegeben haben. Dies bedeutet aber nicht, dass sexualisierte Gewalt nicht auch in vielen anderen Institutionen und Bereichen ein erhebliches Problem darstellt [3].

## Fazit für die Praxis

- Therapeutinnen und Therapeuten sollten wissen, dass sexualisierte Gewalt im Verantwortungsbereich der EKD in der Vergangenheit vorgekommen ist und weiter vorkommt.
- Sexualisierte Gewalt ist aber nicht nur ein Problem der beiden großen christlichen Kirchen, sondern ereignet sich auch in vielen anderen Institutionen und Bereichen, insbesondere auch im familiären Kontext.
- Viele Betroffene leiden unter langanhaltenden psychischen Störungen und sozialen Folgen und bedürfen einer psychotherapeutischen Unterstützung bei der Aufarbeitung der Missbrauchserlebnisse.
- Betroffene sollten ermutigt werden, über ihre Erfahrungen zu berichten und ihre Ansprüche gegenüber der Kirche und dem Staat geltend zu machen.

## References

[1] forum-studie.de. Abschlussbericht_ForuM_21-02-2024.pdf. Zugegriffen: 25. März 2024

[2] Dreßing H, Dölling D, Hermann D, Kruse A, Schmitt E, Bannenberg B, Hoell A, Voss E, Salize HJ (2019) Sexual abuse at the hands of catholic clergy. Dtsch Ärztebl Int 116(22):389–396. 10.3238/arztebl.2019.0389 31366429 10.3238/arztebl.2019.0389 PMC6676731

[3] Dreßing H, Dölling D, Hermann D, Horten B, Kruse A, Schmitt E, Bannenberg B, Whittaker K, Salize HJ (2017) Sexual abuse of minors within the Catholic Church and other institutions : A literature review. Neuropsychiatr 31(2):45–55 28405901 10.1007/s40211-017-0223-4

[4] EKD: Sichten von Personalakten für Studie war vereinbart. evangelisch.de. Zugegriffen: 25. März 2024

[5] Vorstellung Studie zu Missbrauch in der evangelischen Kirche. youtube.com (Erstellt: 25. Jan. 2024). Zugegriffen: 25. März 2024

